# Help-Seeking Intentions for Depression and Associated Factors among Chinese Perinatal Women: A Cross-Sectional Study

**DOI:** 10.3390/ijerph20032288

**Published:** 2023-01-27

**Authors:** Sasa Huang, Ying Hu, Bing Fu, Guanxiu Tang, Zhihong Chen, Lijuan Zhang, Meili Xiao, Jun Lei

**Affiliations:** 1 Nursing Department, The Third Xiangya Hospital of Central South University, 138 Tongzipo Road, Yuelu District, Changsha 410013, China; 2 Xiang Ya Nursing School, Central South University, 172 Tongzipo Road, Yuelu District, Changsha 410013, China; 3 Department of Obstetrics and Gynecology, The Third Xiangya Hospital of Central South University, 138 Tongzipo Road, Yuelu District, Changsha 410013, China; 4 Department of Geriatrics, The Third Xiangya Hospital of Central South University, 138 Tongzipo Road, Yuelu District, Changsha 410013, China; 5 Department of Pediatric Intensive Care Unit, The Third Xiangya Hospital of Central South University, 138 Tongzipo Road, Yuelu District, Changsha 410013, China; 6 Department of Emergency Pediatrics, The Third Xiangya Hospital of Central South University, 138 Tongzipo Road, Yuelu District, Changsha 410013, China

**Keywords:** help-seeking intention for depression, perinatal depression, help-seeking attitude, stigma, mental health literacy

## Abstract

A low help-seeking intention for depression is an important reason for the low number of women with perinatal depression who have sought professional help. However, evidence of help-seeking intentions for depression is still lacking in Chinese perinatal women. We aimed to investigate the help-seeking intention for depression and its associated factors among Chinese perinatal women. Participants were recruited from three comprehensive hospitals in Changsha. A total of 874 perinatal women were included in the study. The score for the help-seeking intention for depression in Chinese perinatal women was 3.65 ± 0.79, with about half of participants (58.3%) reporting that they were “likely” and “strongly likely” to seek professional help if they suffered from depression during the perinatal period. Favorable help-seeking attitudes and sufficient knowledge of mental illness help-seeking resources were positively associated with help-seeking intentions for depression. However, self-stigma decreased the help-seeking intention for depression. Chinese perinatal women had relatively positive help-seeking intentions for depression. Reducing the stigma of mental illness and help-seeking, enhancing mental health literacy, and improving attitudes toward professional psychological help-seeking of perinatal women may be the potential key components of interventions to encourage perinatal women to actively seek professional psychological help.

## 1. Introduction

Perinatal depression (PND) is a common perinatal complication with different degrees of depressive symptoms occurring during pregnancy or within the first 12 months after birth [1,2]. A recent review of 96 studies showed that the prevalence of perinatal depression was 11.4% in high-income countries and 13.1% in low-and middle-income countries [3]. In China, the prevalence of perinatal depression increased in the last few decades, with 19.7% of cases occurring antenatally and 14.8% postnatally [4]. Perinatal depression has a profound negative impact on maternal and their infants’ health, causing complications such as premature delivery, impairment of the children’s cognitive, emotional, behavioral, and social development, poor mother-infant interaction and family functioning, and even leading to maternal suicide or infanticide [2,5,6].

The profound and negative consequences of perinatal depression indicate that early identification and treatment are significant for women with perinatal depression. However, previous studies showed that the majority of women with perinatal depression sought less psychological help or delayed help-seeking [7,8,9] even though there are effective treatments for perinatal depression [10,11]. Studies conducted in the United States reported that only 13.6–18.1% of women who screened positive for depression sought professional help for depressive symptoms [7,8]. In Ethiopia, 12.7% of postpartum women sought help from professionals for their depressive symptoms [9]. In the Middle East, postpartum women had less frequent behaviors toward professional psychological help-seeking, with around only 4% of postpartum women consulting professional assistance [12].

According to the theory of planned behavior, the intention to seek help is the strongest predictor of actual help-seeking behavior; the stronger the positive help-seeking intention, the more likely an individual is to seek professional help [13,14], indicating that promoting the help-seeking intention is the critical step to promote help-seeking behavior. A study in Chinese perinatal women indicated that women with stronger help-seeking intentions were more likely to seek professional psychological help [15]. Moreover, studies conducted in other populations (e.g., young people and older Australians with depressive symptoms) also revealed that a help-seeking intention was a positive predictor of help-seeking behavior [16,17]. Therefore, understanding the help-seeking intention for depression and its associated factors in perinatal women is significant for early identification of those women who are at high risk of a low likelihood of seeking professional help and developing targeted interventions to improve their professional psychological help-seeking behaviors. Previous studies have reported that various factors could affect the intention of perinatal women to seek professional psychological help, such as younger age, low-level education, living in an urban area, first-time birth, a history of mental illness and professional psychological help-seeking behaviors [18,19,20,21], the severity of depressive symptoms [9], mental health literacy [7], and stigma and negative attitudes towards help-seeking [22]. However, current studies focused on perinatal women’s help-seeking intentions for depressive symptoms are mainly explored in developed countries (e.g., US and Israel), and very little is known about help-seeking intentions for depression among perinatal women in China. Studies have shown that there exists a difference in an individual’s help-seeking intention in different cultural contexts [23]. Therefore, studies of help-seeking intentions for depression in Chinese perinatal women are still needed.

To our knowledge, only one study has provided some important views on help-seeking intention in Chinese pregnant women with probable depression or anxiety during the COVID-19 pandemic period [21], and it found that only 19% of Chinese pregnant women had the intention to seek help for depression or anxiety. However, this finding only found some COVID-19-related factors (e.g., COVID-19-related lockdowns in the residence area, perceived social support during the COVID-19 pandemic, and trust in health care providers) associated with Chinese pregnant women’s intentions to seek mental health services. Moreover, maternal age and education level, which have been reported to be associated with the help-seeking intention for depression in previous studies [7,24,25], were found to be correlated with having no intention to seek help. In addition, significant factors (e.g., the severity of depressive symptoms, help-seeking attitude, stigma, and mental health literacy) that have been found to play an important role in perinatal women’s help-seeking processes have not been studied in this study. Therefore, further exploration of perinatal women’s help-seeking intentions for depression in the Chinese cultural background is necessary.

A clear understanding of the factors impacting help-seeking intentions for depression among perinatal women would contribute to developing some public health strategies and significantly positively impact their help-seeking behaviors within the Chinese cultural background. Therefore, this study aims (1) to explore help-seeking intention for depression among Chinese perinatal women and (2) to identify factors associated with the help-seeking intention for depression in this sample.

## 2. Materials and Methods

### 2.1. Procedure and Participants

A population-based cross-sectional study was used in this study. Participants were recruited from three comprehensive hospitals in Changsha, Hunan Province, China through a convenience sampling technique. Women who took part in prenatal check-ups or routine postpartum examinations 42 days after delivery were contacted by research assistants in the obstetric clinics of three comprehensive hospitals. The eligible criteria for participants were as follows: (1) more than 18 years old; (2) women in any trimester during pregnancy or within the period from birth to 12 months postpartum. Participants without the intention to continue the pregnancy, those who had terminated the pregnancy, or those who had a stillbirth were excluded. A total of 950 women were initially invited based on eligible criteria, of which 890 women agreed to participate in this study and completed the questionnaires (acceptance rate: 93.7%). Finally, a total of 874 questionnaires were validated, with 16 questionnaires excluded due to having missing values (effective callback rate: 98.2%). 

Before starting the survey, participants were informed about the survey design, purpose, and contents, and that their personal information would be kept confidential. All participants consented to sign informed consent forms and spent about 15–20 min to complete the questionnaires. This study was approved by the Xiang Ya Nursing School of Central South University (No. E202222).

### 2.2. Measures

#### 2.2.1. Demographic Characteristics

Participants’ demographic information included: age, perinatal period, monthly household income (Yuan), residence, parity, education level, spouse’s education level, employment status, spouse’s employment status, relationship with husband or partner (satisfied or unsatisfied), pregnancy-related complications (yes or no), history of mental illness (yes or no), and history of professional psychological help-seeking behaviors (yes or no).

#### 2.2.2. Help-Seeking Intention for Depression

Participants’ help-seeking intentions for depression were measured based on one question, which was adapted from other relevant studies [26]. Participants were specially asked, “When you suffer from depression during the perinatal period, how likely would you choose to seek professional psychological help?” with answers in the form of a 5-point Likert scale scoring from 1 (strongly unlikely) to 5 (strongly likely), with higher scores indicating a more positive help-seeking intention for depression. A score greater than 3 indicated a positive help-seeking intention for depression, while a score less than 3 indicated a negative help-seeking intention for depression.

#### 2.2.3. Depressive Symptoms

The Edinburgh Postnatal Depression Scale (EPDS), a 10-item self-report scale, was used to assess perinatal women’s depressive symptoms in the past seven days [27]. Each item on the EPDS was responded to with a 4-point Likert scale, scoring from 0 (“never”) to 3 (“often”). The total score of the EPDS ranges from 0 to 30, with higher scores indicating more serious depressive symptoms. The EPDS demonstrated good internal consistency (Cronbach’s α was 0.87) in the study conducted by Cox et al. [27]. The Cronbach’s α coefficient for this scale was 0.82 in our sample.

#### 2.2.4. Attitude toward Seeking Professional Psychological Help

The Chinese version of the Attitudes Toward Seeking Professional Psychological Help Scale-Short Form (ATSPPH-SF) was used to assess participants’ attitudes toward seeking professional psychological help [28]. ATSPPH-SF includes 10 items and 2 subscales: openness to seeking professional help for emotional problems (items 1, 3, 5, 6, 7) and value and need in seeking professional help (items 2, 4, 8, 9, 10). Each item of ATSPPH-SF uses a 4-point Likert scale scoring from 0 (“strongly disagree”) to 3 (“strongly agree”). The total score of ATSPPH-SF ranges from 0 to 30, with higher scores indicating more positive attitudes toward seeking professional psychological help. According to Fisher et al. [29], the ATSPPH-SF demonstrated good internal consistency. In our sample, Cronbach’s α coefficient for the scale and two subscales were 0.72, 0.77, and 0.65, respectively.

#### 2.2.5. Stigma for Seeking Professional Psychological Help

The Questionnaire of Stigma for Seeking Professional Psychological Help (SSPPH) was used to assess participants’ perceived stigma towards seeking help for depression [30]. The SSPPH includes 10 items and two subscales: self-stigma and public stigma of seeking professional psychological help. Each item of the SSPPH is rated on a 5-point Likert scale, scoring from 1 (strongly disagree) to 5 (strongly agree). The SSPPH scores range from 10 to 50, with higher scores representing increased self-stigma and public stigma for seeking professional psychological help. The SSPPH scale demonstrated good internal consistency (Cronbach’s α was 0.81) [30]. In our sample, Cronbach’s α coefficient for this scale and two subscales were 0.94, 0.91, and 0.90, respectively.

#### 2.2.6. Mental Health Literacy

Participants’ perception of knowledge and beliefs about mental illness was measured using the Multicomponent Mental Health Literacy Measure, which was translated by Chinese scholar Zhijun Ming [31]. It consists of 22 items with 3 subscales: knowledge-oriented MHL (10 items), beliefs-oriented MHL (8 items), and resource-oriented MHL (4 items). The response format of the first 18 items uses six responses: “strongly disagree”, “disagree”, “neutral”, “agree”, “strongly agree”, and “I don’t know”. Each response for each item was grouped into one of two categories. For knowledge-oriented MHL items, participants who answered “strongly agree” and “agree” would be coded 1; those who answered “strongly disagree”, “disagree”, “neutral”, and “I don’t know” would be coded 0. For beliefs-oriented MHL items, participants who answered “strongly disagree” and “disagree” would be coded 1, and those who answered “strongly agree”, “agree”, “neutral”, and “I don’t know” would be coded 0. For resource-oriented MHL items, the response format was rated on a dichotomous scale: “yes (coded 1)” or “no (coded 0)”. The total score ranges from 0 to 22, with a higher score indicating better mental health literacy. The scale demonstrated good internal consistency (Cronbach’s α was 0.83) in the study conducted by Jun et al. [32]. In our sample, Cronbach’s α coefficient for this scale and the three subscales were 0.86, 0.86, 0.81, and 0.83, respectively.

### 2.3. Statistical Analyses

Data analysis was performed using Statistical Product and Service Solutions version 22.0. Descriptive statistics was used to explore the participants’ characteristics, related variables (depressive symptoms, help-seeking attitude, stigma, and mental health literacy), and the outcome variable (help-seeking intention for depression). Continuous variables were presented as mean and standard deviation, and categorical variables were presented as frequency and percentage. In this study, an independent Student’s t-test and a one-way ANOVA test were used to examine the effect of demographic variables on the help-seeking intention for depression; the correlation analysis was used to explore the relationship between depression, help-seeking attitude, stigma, mental health literacy, and help-seeking intention for depression. A multiple linear regression analysis was performed to explore the significantly correlated factors associated with the help-seeking intention for depression in perinatal women, and a two-sided *p*-value < 0.05 was considered a statistically significant association.

## 3. Results

### 3.1. Participants’ Characteristics

A total of 874 perinatal women were included in this study. The maternal characteristics are presented in Table 1. In this study, most of the participants were <35 years old (*n* = 730, 83.5%), lived in urban area (*n* = 764, 87.4%), presented a bachelor’s degree or above (*n* = 716, 81.9%), and had a satisfactory relationship with their husbands or partners (*n* = 821, 93.9%). More than half of the participants were in their first pregnancy (*n* = 503, 57.6%), had a monthly household income of over 8000 Yuan (*n* = 529, 60.5%), and were employed (*n* = 566, 64.8%). In addition, a small number of participants had pregnancy-related complications (*n* = 218, 24.9%), a history of mental illness (*n* = 21, 2.4%), and a history of professional psychological help-seeking behaviors (*n* = 11, 1.3%).

### 3.2. Perinatal Women’s Help-Seeking Intentions for Depression

**Figure 1:**
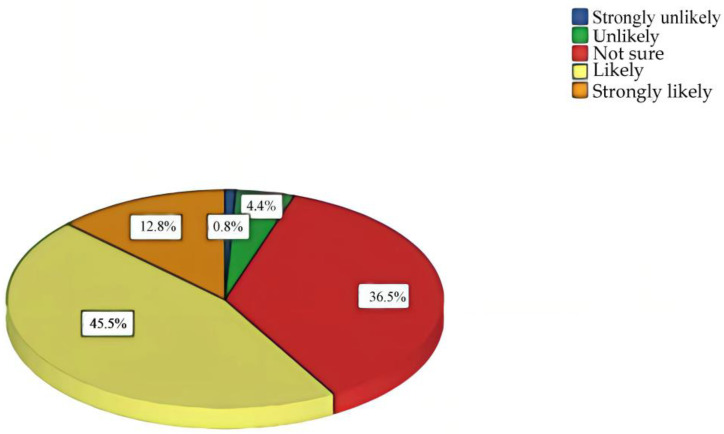
Help-seeking intentions for depression among Chinese perinatal women (*n* = 874).

The average score of help-seeking intention for depression among Chinese perinatal women was 3.65 ± 0.79, with 3.65 ± 0.78 antenatally and 3.67 ± 0.81 postnatally. Nearly half of the participants (58.3%) reported that they would be “likely” and “strongly likely” to seek professional psychological help if they suffered from depression during the perinatal period (see Figure 1). The findings in this study indicated that Chinese perinatal women had relatively positive help-seeking intentions for depression.

### 3.3. Association between Maternal Characteristics and Help-Seeking Intentions for Depression

In the univariable analysis (see Table 2), women who had higher household income (Yuan), had a higher education level, were primipara, were in employment status, and had a history of mental illness were related to higher help-seeking intentions for depression (*p* < 0.05). Moreover, those women whose spouses had higher education levels and were employed were significantly associated with higher help-seeking intentions for depression (*p* < 0.05).

### 3.4. Correlations between Depression, Help-Seeking Attitude, Stigma, Mental Health Literacy, and Help-Seeking Intentions for Depression

Table 3 shows that the help-seeking intention for depression was significantly positively associated with openness to seeking professional help for emotional problems (*r* = 0.244, *p* < 0.001), value and need in seeking professional help (*r* = 0.376, *p* < 0.001), knowledge-oriented MHL (*r* = 0.193, *p* < 0.001), beliefs-oriented MHL (*r* = 0.200, *p* < 0.001), and resource-oriented MHL (*r* = 0.194, *p* < 0.001). However, help-seeking intention for depression was negatively correlated with a higher score of depression (*r* = −0.131, *p* < 0.001), self-stigma (*r* = −0.284, *p* < 0.001), and public stigma (*r* = −0.263, *p* < 0.001).

### 3.5. Multiple Linear Regression of Factors Associated with Help-Seeking Intention for Depression

The results of multiple linear regression (seen in Table 4) show that depressive symptoms and maternal characteristics did not contribute to help-seeking intentions for depression. Perinatal women who had a more positive attitude toward openness to seeking professional help for emotional problems (β = 0.095, *p* = 0.004), a more positive attitude toward the value and need in seeking professional help (β = 0.343, *p* < 0.001), and sufficient knowledge of resource-oriented MHL (β = 0.068, *p* = 0.031) were significantly associated with increased help-seeking intentions for depression. However, perinatal women with more self-stigma were more likely to have lower help-seeking intentions for depression (β = −0.129, *p* < 0.001).

## 4. Discussion

In this study, we found that Chinese perinatal women had relatively positive help-seeking intentions for depression. Women with a more positive attitude toward seeking professional psychological help and sufficient knowledge of help-seeking resources for mental illness were positively associated with increased intentions to seek psychological help for depression. However, higher self-stigma was associated with lower help-seeking intentions for depression.

### 4.1. Perinatal Women’s Help-Seeking Intentions for Depression

In our study, the mean score of the help-seeking intention for depression among perinatal women was 3.65 ± 0.79, implying that Chinese perinatal women have relatively positive help-seeking intentions for depression. This finding was similar to the results from Portugal using the same tool but with a different range: from one (extremely unlikely) to seven (extremely likely) [33]. In line with a Portuguese study [7], our results also indicated that most perinatal women (58.3%) would likely seek help from professionals if they were suffering from depression. Moreover, we found that different assessment tools to measure help-seeking intentions for depression (e.g., five-point Likert scale, seven-point Likert scale, or a scale with a dichotomy method of scoring) were used in current studies, which resulted in great heterogeneity in the incidence of perinatal women’s help-seeking intentions. Therefore, a standardized assessment instrument for help-seeking intentions should be conducted in future studies, which would be helpful for data aggregation and outcome comparisons among different studies as well as a cross-cultural discussion of help-seeking intentions for depression.

### 4.2. Factors Associated with Perinatal Women’s Help-Seeking Intentions for Depression

#### 4.2.1. Demographic Factors and Depressive Symptoms

The current study found that demographic characteristics were not significant predictors of Chinese perinatal women’s help-seeking intentions for depression, which contrasts with previous studies that revealed the associations between some demographic characteristics (e.g., older age, high-level education) and perinatal women’s help-seeking intentions for depression [18,19,21]. Moreover, in contrast to previous studies that have revealed that an increased severity of depressive symptoms increases one’s help-seeking intention for depression [9,34], our study found that the severity of depressive symptoms was not associated with the help-seeking intention for depression among Chinese perinatal women. Chinese perinatal women’s demographic factors and depressive symptoms might have an indirect influence on the help-seeking intention for depression through other variables (e.g., help-seeking attitude and stigma). For example, a study of mental healthcare utilization among German community populations reported that some demographic characteristics (e.g., female gender and older age) could positively affect help-seeking intention by exerting a negative effect on the stigma of mental illness [24]. Another study in Chinese community-dwelling populations found that depressive symptoms could influence help-seeking intention indirectly by the mediating effect of stigma and help-seeking attitudes [35]. Above all, our findings indicated that the relationship between demographic characteristics, depressive symptoms, and the help-seeking intention for depression in Chinese perinatal women is complex and has not been well-studied. More structural equation modeling analysis-related studies are needed to further explore the effect mechanism of demographic factors, depressive symptoms, and other factors on Chinese perinatal women’s help-seeking intentions for depression, which could provide valuable basic data to develop target interventions to improve the help-seeking behaviors and mental health of perinatal women.

#### 4.2.2. Attitudes toward Seeking Professional Psychological Help

In this study, we found that those perinatal women who had a more positive help-seeking attitude were more likely to seek help from professionals when they suffered from depressive symptoms during the perinatal period, which was consistent with previous studies [36,37,38]. Help-seeking attitude is defined as an individual’s perception and evaluation of seeking professional psychological help (e.g., the perceived need for psychological help, the trust for professionals, the confidence in psychological help, and the concern of psychological problems) [39]. Help-seeking intention refers to the possibility or willingness of an individual to seek professional psychological help [40]. Studies have shown that perinatal women with a more positive help-seeking attitude often had more confidence in mental health professionals and less self-stigma associated with mental illness, which increased their motivation and willingness to seek professional help [41,42]. Ajzen’s study also supported this finding, which confirmed that individuals with a more positive help-seeking attitude have stronger intentions to seek professional help [13]. Therefore, to promote those at high risk of low likelihood to receive mental health services, improving women’s attitudes toward seeking professional psychological help is necessary.

#### 4.2.3. Stigma

Our study found that perinatal women who perceived more self-stigma were more likely to report negative intentions to seek help from professionals for depression. This finding was consistent with previous studies [43,44,45,46]. According to cultural and personal beliefs about motherhood and the role of women, a good mother should be “strong”, be able to handle everything, and should not be depressed [12,47,48]. This high expectation of women to be “good mothers” could result in women with self-stigma when they suffer from depression and choose negative coping strategies (e.g., secrecy, avoidance, and concealing inner feelings) to deal with emotional problems, as they would feel shame or guilt to talk about psychological distress with professionals [49,50,51]. In addition, mental illness is usually linked with a sign of “sickness”, “incompetence”, and “weakness” in Chinese traditional culture, and individuals would not seek any professional psychological help for their “face”, “reputation”, and “family harmony”. This cultural prejudice makes Chinese people prefer to cope with mental health problems by themselves and perceive self-stigma when they disclose mental health problems to others, resulting in their reluctance to seek help from professionals [24,42,52,53]. Accordingly, it can be concluded that the stereotypes of motherhood and mental illness in culture enhance the self-stigma for seeking professional psychological help, resulting in low help-seeking intentions for depression. This highlights that there is an urgent need, both in China and other countries, to develop a de-stigmatizing strategy based on cultural background.

#### 4.2.4. Mental Health Literacy

Mental health literacy (MHL) is described as “knowledge and beliefs toward mental health that facilitate recognition, management, and prevention of mental disorders” [54], which could reflect individuals’ understanding of the signs and symptoms of mental disorders and the need for professional help. We found that sufficient knowledge of help-seeking resources for mental illness was significantly associated with more positive help-seeking intentions for depression among Chinese perinatal women; however, no associations were found between knowledge and belief of mental illness and help-seeking intentions for depression in the current study. This finding was similar to studies conducted in India and Iran, which reported that the dimension of knowledge of how to seek information related to postpartum depression or professional help scored lower than other dimensions [55,56]. Furthermore, previous studies also suggested that women with inadequate knowledge of help-seeking resources or professional help services available usually did not know which mental care services were available to them, where they could go to obtain professional psychological help, and even had difficulty in identifying the accuracy of information about the available resources for perinatal depression through informal sources (e.g., internet, TV, friends, and family members), all of which could decrease their motivation and need to seek professional psychological help and even cause some of them to adopt harmful self-help measures (e.g., drinking alcohol or smoking marijuana) to deal with their distress [47,49,56,57,58]. Consequently, there is an urgent need to improve the awareness of perinatal depression through various educational programs, such as delivering knowledge of perinatal depression, available professional help resources, and mental health services in community campaigns and pregnancy schools. In addition, the cross-sectional nature of the study indicates that help-seeking intentions for depression could maybe drive perinatal women to seek knowledge of MHL resources, which highlights that more studies are needed to explore the effect mechanism between help-seeking intentions and knowledge of MHL resources.

### 4.3. Implications

The findings of our study have some significant implications to improve the help-seeking intention for depression among Chinese perinatal women. First, the help-seeking intention for depression among Chinese perinatal women should be explored further in future research, and an understanding of the mechanism of how some influencing factors work together to predict women’s help-seeking intentions for depression is needed, which could provide the theoretical reference to develop targeted interventions to promote mental health service utilization against perinatal depression. Second, a standardized assessment instrument for help-seeking intention is needed, which is helpful to assess perinatal women’s help-seeking intentions for depression more accurately and develop effective interventions. Third, mental health literacy and help-seeking attitude play an important role in improving help-seeking intentions for depression among Chinese perinatal women. The theory of knowledge–attitude–practice suggests that knowledge is the first step to developing a positive attitude and promoting behavioral change [59]. And such education should include perinatal depression-related knowledge (e.g., symptom identification, risk factors, damage, treatment options, depression screen, and attitudes facilitating appropriate professional help-seeking), especially regarding paying more attention to information about available mental health services. Finally, de-stigmatizing strategies or interventions (e.g., open discussion of perinatal depression in society, carrying out public health campaigns about perinatal depression, and encouraging mental health providers to avoid using stigmatizing language or to use non-threatening, non-judgmental communication techniques with women) should be considered in clinical practice because self-stigma was a negative prediction factor for perinatal women’s help-seeking intentions for depression.

### 4.4. Strengths and Limitations

This study was the first study that included a relatively large sample size to explore help-seeking intentions for depression in Chinese perinatal women during the normalized COVID-19 epidemic mode.

This study also has several limitations. First, our study has some limitations on generalizability because we only included participants from one region in Changsha, Hunan, and the majority of participants were of a younger age, had higher education, and lived in urban areas. Second, we only assessed participants’ help-seeking intentions for depression, participants’ intentions to seek help for depression from different professionals were not investigated, which would be beneficial to understand perinatal women’s help-seeking preferences and develop targeted interventions. Third, the cross-sectional results in our study emphasize that it is necessary to measure perinatal women’s help-seeking intentions or actual professional help-seeking behaviors for depression through longitudinal studies to more accurately explore predictive factors of help-seeking for depression. Finally, a single item measuring help-seeking intentions for depression was used in this study. However, it should be noted that a single item to measure help-seeking intentions for depression cannot fully represent a complex construct. Moreover, using a single item to measure help-seeking intentions for depression in this study is problematic and has low content validity and reliability. Therefore, an increased measurement scale of the intention to seek professional psychological help for depression is needed in further studies.

## 5. Conclusions

Chinese perinatal women’s help-seeking intentions for depressive symptoms during the perinatal period were relatively positive. A positive help-seeking attitude and sufficient knowledge of help-seeking resources for mental illness were facilitators of perinatal women’s help-seeking intentions for depression. However, self-stigma was a barrier to perinatal women’s help-seeking intentions for depression. Interventions for increasing awareness about perinatal depression and attitudes toward help-seeking, and stigma-reduction campaigns should be provided to improve Chinese perinatal women’s help-seeking intentions for depression.

## Figures and Tables

**Table 1:** The characteristics of participants (*n* = 874).

Sociodemographic Characteristics	*n*	%
Age (years)		
<35	730	83.5
≥35	144	16.5
Perinatal period		
Prenatal	753	86.2
Postpartum	121	13.8
Monthly household income (Yuan)		
<4000	51	5.9
4000–6000	112	12.8
6001–8000	182	20.8
>8000	529	60.5
Residence		
Urban	764	87.4
Rural	110	12.6
Parity		
Primipara (nulliparous pregnant women)	503	57.6
Multiparous (women who have given birth)	371	42.4
Education level		
High school or below	158	18.1
Bachelor’s degree	632	72.3
Postgraduate or above	84	9.6
Spouse’s education level		
High school or below	184	21.0
Bachelor’s degree	595	68.1
Postgraduate or above	95	10.9
Employment status		
Unemployed status	308	35.2
Employed status	566	64.8
Spouse’s employment status		
Unemployed status	51	5.8
Employed status	823	94.2
Relationship with husband or partner		
Satisfied	821	93.9
Unsatisfied	53	6.1
Pregnancy-related complications		
No	656	75.1
Yes	218	24.9
History of mental illness		
No	853	97.6
Yes	21	2.4
History of professional psychological help-seeking behaviors		
No	863	98.7
Yes	11	1.3

**Table 2:** Comparison of the help-seeking intention for depression in perinatal women (*n* = 874).

Sociodemographic Characteristics	M (SD)	t/F	*p*
Age (years)		1.847	0.065
<35	3.67 (0.78)		
≥35	3.54 (0.82)		
Perinatal period		−0.260	0.795
Prenatal	3.65 (0.78)		
Postpartum	3.67 (0.81)		
Monthly household income (Yuan)		2.739	0.042
<4000	3.63 (0.94)		
4000–6000	3.63 (0.75)		
6001–8000	3.52 (0.74)		
>8000	3.71 (0.79)		
Residence		−1.133	0.257
Urban	3.66 (0.78)		
Rural	3.57 (0.83)		
Parity		2.176	0.030
Primipara (nulliparous pregnant women)	3.70 (0.79)		
Multiparous (women who have given birth)	3.58 (0.79)		
Education level		7.403	0.001
High school or below	3.50 (0.84)		
Bachelor’s degree	3.66 (0.77)		
Postgraduate or above	3.90 (0.79)		
Spouse’s education level		6.365	0.002
High school or below	3.48 (0.82)		
Bachelor’s degree	3.68 (0.77)		
Postgraduate or above	3.80 (0.80)		
Employment status		−2.698	0.007
Unemployed status	3.56 (0.83)		
Employed status	3.70 (0.76)		
Spouse’s employment status		−2.069	0.039
Unemployed status	3.43 (0.96)		
Employed status	3.67 (0.77)		
Relationship with husband or partner		−1.725	0.085
Satisfied	3.66 (0.77)		
Unsatisfied	3.47 (0.95)		
Pregnancy-related complications		0.216	0.829
No	3.66 (0.79)		
Yes	3.64 (0.79)		
History of mental illness		−2.055	0.040
No	3.64 (0.79)		
Yes	4.00 (0.55)		
History of professional psychological help-seeking behaviors		−1.477	0.140
No	3.65 (0.79)		
Yes	4.00 (0.63)		

**Table 3:** Correlations between depression, help-seeking attitude, stigma, mental health literacy, and help-seeking intention for depression (*n* = 874).

Variables	1.	2.	3.	4.	5.	6.	7.	8.
1. Depression	1							
2. Openness to seeking professional help for emotional problems	−0.184 **	1						
3. Value and need in seeking professional help	−0.132 **	0.195 **	1					
4. Self-stigma	0.364 **	−0.364 **	−0.274 **	1				
5. Public-stigma	0.256 **	−0.328 **	−0.255 **	0.688 **	1			
6. Knowledge-oriented MHL	−0.095 **	0.125 **	0.240 **	−0.235 **	−0.226 **	1		
7. Beliefs-oriented MHL	−0.139 **	0.332 **	0.175 **	−0.289 **	−0.291 **	0.327 **	1	
8. Resource-oriented MHL	−0.168 **	0.188 **	0.236 **	−0.222 **	−0.177 **	0.242 **	0.168 **	1
9. Help-seeking intention for depression	−0.131 **	0.244 **	0.376 **	−0.284 **	−0.263 **	0.193 **	0.200 **	0.194 **

** *p* < 0.001 (two tailed).

**Table 4:** Multivariable analysis of factors associated with the help-seeking intention for depression (*n* = 874).

Variables	B	SE	β	*p*	95%CI
Openness to seeking professional help for emotional problems	0.035	0.012	0.095	0.004	0.011, 0.059
Value and need in seeking professional help	0.125	0.012	0.343	<0.001	0.102, 0.149
Self-stigma	−0.030	0.008	−0.129	<0.001	−0.046, 0.015
Resource-oriented MHL	0.033	0.015	0.068	0.031	0.003, 0.064

B: unstandardized coefficients; SE: standard error; β: standardized coefficients; 95%CI: 95% confidence interval.

## Data Availability

The data generated during the present study are available from the corresponding author upon reasonable request.
